# Unfakeable Facial Configurations Affect Strategic Choices in Trust Games with or without Information about Past Behavior

**DOI:** 10.1371/journal.pone.0034293

**Published:** 2012-03-28

**Authors:** Constantin Rezlescu, Brad Duchaine, Christopher Y. Olivola, Nick Chater

**Affiliations:** 1 Cognitive, Perceptual and Brain Sciences, University College London, London, United Kingdom; 2 Department of Psychological and Brain Sciences, Dartmouth College, Hanover, New Hampshire, United States of America; 3 Warwick Business School, University of Warwick, Coventry, United Kingdom; University of Minnesota, United States of America

## Abstract

**Background:**

Many human interactions are built on trust, so widespread confidence in first impressions generally favors individuals with trustworthy-looking appearances. However, few studies have explicitly examined: 1) the contribution of unfakeable facial features to trust-based decisions, and 2) how these cues are integrated with information about past behavior.

**Methodology/Principal Findings:**

Using highly controlled stimuli and an improved experimental procedure, we show that unfakeable facial features associated with the appearance of trustworthiness attract higher investments in trust games. The facial trustworthiness premium is large for decisions based solely on faces, with trustworthy identities attracting 42% more money (Study 1), and remains significant though reduced to 6% when reputational information is also available (Study 2). The face trustworthiness premium persists with real (rather than virtual) currency and when higher payoffs are at stake (Study 3).

**Conclusions/Significance:**

Our results demonstrate that cooperation may be affected not only by controllable appearance cues (e.g., clothing, facial expressions) as shown previously, but also by features that are impossible to mimic (e.g., individual facial structure). This unfakeable face trustworthiness effect is not limited to the rare situations where people lack any information about their partners, but survives in richer environments where relevant details about partner past behavior are available.

## Introduction

The temptation to judge strangers by their faces is hard to resist. Although many of us believe we can tell the virtuous from the wicked by their faces [1], research suggests there is limited value in face-based judgments [1], [2] (although see [3], [4]). Overtly, we label the use of this self-perceived ability as unethical; nevertheless, the fast and spontaneous process [5] of inferring traits from faces influences a wide range of consequential decisions (e.g., [6] [7] [8] [9]).

Among the various traits inferred from faces, trustworthiness is one of the most important for social and economic interactions. Trust, a true “lubricant of a social system” [10], pervades all economic exchanges [11]; investments and partnerships could not occur without it. Furthermore, people generally agree on who looks trustworthy [12], suggesting a potential generalized tendency to trust certain individuals (those with the “right” face), all other things being equal.

However, subjective ratings of perceived trustworthiness may not translate into behavior. Our study examines whether people take potentially costly actions in line with their face-based trustworthiness judgments. Recent studies have found that appearance-based perceptions of borrower trustworthiness predict lending tendencies in online peer-to-peer lending, even when lenders have demographic and financial information about borrowers (Duarte, J., Siegel, S., & Young, L. A. *Trust and credit*. American Finance Association Annual Meeting 2010, Atlanta, retrieved April 1, 2011, from SSRN: http://ssrn.com/abstract=1343275; Ravina, E. *Beauty, personal characteristics and trust in credit markets*. American Law & Economics Association Annual Meeting 2008, Stanford, retrieved April 1, 2011, from http://law.bepress.com/alea/18th/art67). But these studies are correlational, and borrower photos often included more than faces, so it is uncertain what aspects of appearances influenced investment choices. Other studies using the controlled environment of trust games [13], have demonstrated a causal role of facial cues, with participants investing more in partners with trustworthy-looking faces [14], [15]. Facial resemblance between investor and trustee [16], facial expression, and an aggregated measure of cooperativeness derived from face evaluations [17] also predicted investment choices.

Despite these suggestive results, important questions remain unanswered. First, what features are evaluated when making decisions based on face trustworthiness judgments? In the absence of reputational information, perhaps we use any cues available concerning an economic counterpart's trustworthiness. However, to be considered reliable, these cues must be difficult to simulate. Since “fakeable” cues, such as hairstyle and clothing, can be manipulated to send false signals, their informational value should be limited. Facial physiognomy, in contrast, provides unfakeable perceptual cues. Yet evidence convincingly demonstrating that unfakeable facial features (which cannot be altered by targets) bias economic choices is scarce. For example, van't Wout and Sanfey's face stimuli [15] varied in hair style, eye gaze, glasses, etc., so trustworthiness judgments and investment decisions could have been based on these changeable dimensions rather than stable facial features. Other authors have considered specific stable facial attributes in isolation: Stirrat and Perret [14] found that wider male faces were rated more trustworthy and attracted more investments in trust games.

Here, we examine whether unfakeable facial features can influence trust in a controlled experimental setting. In contrast to Stirrat and Perret [14], our approach is holistic: rather than focusing on a single feature (e.g., facial width ratio), our face stimuli vary on a multi-dimensional physiognomic space, along directions previously shown to correlate with perceived trustworthiness but not perceived dominance [12]. These stimuli were generated by an empirically validated computer-based model of trustworthiness that manipulates normally stable facial features. The faces are standardized, with no hair, no facial marks or other specific identifiers, have the same skin texture, and neutral expressions [18]. In our first experiment, participants decide, in a series of trust games, how much to invest in various trustees represented by these computerized faces. Because our stimuli are tightly controlled, any observed differences in investments can only be due to stable facial configurations subjectively associated with trustworthiness.

A second unanswered question is whether the effects of trustworthy-looking facial configurations survive in richer informational environments. Most prior experimental studies offered participants no information about their partners beyond their faces, a situation rarely encountered in real life [2]. People usually have access to information about prospective partners beyond their appearances and face judgments are known to be quickly updated in line with this information [19]. For example, in trust games involving multiple interactions with the same trustee, participants dynamically tuned their investment strategies to favor partners who reciprocated their trust [20]. Across 15 repeated interactions, the main effect of facial trustworthiness was not significant, but trustworthy-looking partners who reciprocated trust still received more money than reciprocating partners with untrustworthy looks. Our second experiment therefore aimed to explore a possible interaction between initial impressions and reputational information. Unlike [20], we used highly controlled facial stimuli to focus on unfakeable facial features. Furthermore, rather than gradually discovering trustee reputations from first-hand interactions, participants saw visual summaries of their partners' past reciprocations (just as one might receive third-party reports about potential business partners). Thus, participants in our second study had simultaneous access to faces and reputational information, so they could integrate both immediately. Finally, participants interacted with each trustee only once, eliminating the potential confound, associated with repeated games, that investment decisions might be used to punish or reward trustees, or to otherwise communicate (dis)satisfaction with a partner's choices [21]. To convey reputational information, we created relatively unambiguous behavioral trustee histories, designed to suggest high or low reciprocity in previous trust games. Rationally, people should focus on trustee past behavior and ignore facial cues. If, on the other hand, participants continue to invest more in trustworthy-looking partners, it shows that the face trustworthiness premium survives even in the presence of reputational information.

Finally, Study 3 provided a replication of our findings under a different incentive scheme, while also controlling for certain artifacts that might have affected our initial results.

## Results and Discussion

### Study 1: Unfakeable facial features

In a series of 40 single-round trust games, played with the Trustworthy and Untrustworthy identities of 20 computer-generated characters, 13 out of 15 participants invested more, on average, in the Trustworthy identities. Using the available range of 0 to 100 virtual pounds (VP), the average amount invested in Untrustworthy identities was 43.69 VP, while Trustworthy identities attracted 61.91 VP (42% more). A 2×20 repeated-measures ANOVA, with invested amount as the dependent variable and character (20 original computer faces) and identity (Untrustworthy vs. Trustworthy) as the two independent variables, revealed a main effect of face identity: F(1, 14) = 12.46, p = .003, partial η^2^ = .47; but not of character: F(19, 266) = 1.56, p = .170 (Greenhouse-Geisser adjusted); and no significant interaction effect: F(19, 266)<1. Thus, manipulating the same character's facial trustworthiness significantly impacted investment decisions, whereas facial differences between the original characters did not.

Study 1 demonstrates unequivocally that stable facial features previously shown to be associated with perceived trustworthiness drive people's investment decisions. Furthermore, it provides supplementary behavioral support for the face trustworthiness model developed by Oosterhof and Todorov [12]. Untrustworthy-looking identities generated by this model attracted smaller investments than their trustworthy-looking counterparts derived from the same original face. This “trustworthiness premium” echoes previous results [14], [15], but also goes a step further by demonstrating, holistically, the influence of unfakeable facial features (those that naturally individuate faces and cannot be deliberately modified, except through cosmetic surgery).

### Study 2: Perceived trustworthiness vs. behavioral history

**Figure 1:**
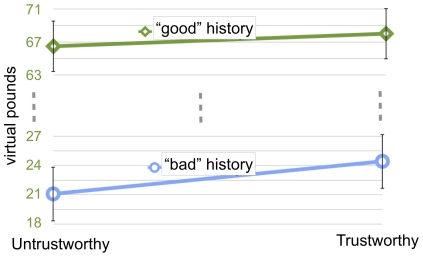
Average amounts invested in Untrustworthy and Trustworthy face identities with “Good” and “Bad” behavioral histories. Note that behavioral histories are represented on two different scales: the blue scale corresponds to Bad history trials; the green scale corresponds to Good history trials. Main effects of both behavioral history and face identity were significant. Error bars represent standard error.

In trust games where reputational information was added next to the trustees' faces, investments were influenced by both histories and face identities (Figure 1). A 2×2 repeated-measures ANOVA, with history (“Bad” and “Good”) and identity (Untrustworthy and Trustworthy) as independent variables, revealed significant main effects of behavioral history: F(1, 51) = 214.48, p<.001, partial η^2^ = .81; and face identity: F(1, 51) = 5.94, p = .018, partial η^2^ = .10, but no interaction effect: F(1, 51) = 2.31, p = .135. The average amount invested in Trustworthy identities (of both “Good” and “Bad” trustees) was 6% higher than the average amount invested in Untrustworthy identities (45.2 versus 42.4 VP).

“Good” histories attracted an average of 67.39 VP, while “Bad” histories attracted an average of 20.65 VP, further confirming that participants considered the colored history matrices to be informative of their partners' tendency to reciprocate. The study also included trials in which participants were only shown the behavioral history of their partner (i.e., without the face presented). This allowed us to compute the facial trustworthiness bonus for each character, which was the difference between the amount invested in the character's trustworthy identity coupled with a behavioral history and the amount invested in the same behavioral history alone, without any face (i.e., facial trustworthiness bonus = trustworthy face−no face). We also computed the facial *un*trustworthiness *penalty* for each character, which was the difference between the amount invested in the character's *un*trustworthy identity coupled with a behavioral history and the amount invested in the same behavioral history alone (i.e., facial untrustworthiness penalty = untrustworthy face−no face). The mean facial trustworthiness bonus, averaged across participants and characters, was 1.57 VP. The mean facial untrustworthiness penalty was −1.83 VP. A within-participant t-test confirmed that the difference between the trustworthiness bonus and the untrustworthiness penalty (Δ = 3.40 VP) was significant: t(51) = 2.94, p = .005, η^2^ = .15. However, a within-participant t-test comparing the absolute values of the trustworthiness bonus and the untrustworthiness penalty failed to reveal a significant difference between the two: t(51) = .16. Thus facial trustworthiness appears to symmetrically shift investments upwards or downwards (relative to no face) according to its valence.

Unfakeable facial features influence economic choices even when people have access to the behavioral histories of their economic partners. While the magnitude of this effect was reduced compared to Study 1, in which faces were presented alone, trustworthy-looking faces were still favored when accompanied by objective cues about trustworthiness. Furthermore, the lack of an interaction between faces and history suggests that the effect is independent of behavioral history type, so that trustees with “Good” and “Bad” histories benefited equally from trustworthy-looking facial features. Finally, trustworthy and untrustworthy identities contributed equally to the facial trustworthiness effect (but in opposite directions).

### Study 3: Trustworthiness premium with higher stakes

Although our first two experiments utilized a virtual currency to circumvent issues associated with the low per-round fees typically paid in multi-round experiments, these low incentives might have failed to motivate serious investment choices.

In our third study we modified the incentive scheme in two ways: (1) by increasing the initial amount that participants could invest on each trial to £5, and (2) by referring to payoffs directly in pounds (£) rather than introducing “virtual pounds” (VP). Since offering £5 each round (with the further possibility of dramatically increasing this amount with each investment) would have been financially impossible in an experiment with multiple trials, we employed a procedure frequently used in the economic literature: participants were given £5 on every round to invest, but the bonus received at the end of the experiment was paid according to the outcome of *only one* randomly selected trial. Therefore, participants had an incentive to treat each trial as if it were a single-shot £5 round.

In contrast to Study 1, in which participants were shown both face versions of each character, in Study 3 we showed participants only one face version of each character, *either* the trustworthy *or* untrustworthy version, with the added constraint that each participant saw 10 trustworthy and 10 untrustworthy faces. Finally, we ensured that trustworthy and untrustworthy faces had a direct eye gaze (the trustworthiness model developed by Oosterhof & Todorov, 2008, causes the eyes in some trustworthy faces to gaze slightly upward and the eyes in some untrustworthy faces to gaze slightly downward).

The results were very similar to those of Study 1. On average, participants invested 40% more in the trustworthy faces than in the untrustworthy faces (£2.8 compared to £2.0). A paired t-test confirmed that this difference was significant: t(19) = 4.80, p<.001. This provides a compelling replication of our initial results and shows that the trustworthiness premium was not the product of insufficient incentives or other methodological artifacts.

## General Discussion

Building on previous studies showing that appearance-based trustworthiness impressions influence cooperation, we examined whether unfakeable facial features perceived to indicate trustworthiness have an impact on economic decisions. Study 1 showed that, when no other cues about a partner's trustworthiness were available, participants invested 42% more in partners with trustworthy-looking facial configurations than in those with untrustworthy-looking faces. This trustworthiness premium was replicated in a follow-up study (Study 3) in which we used higher monetary incentives and controlled for other variables that might have affected our initial results. Study 2 extended the validity of these findings by showing that trustworthy-looking facial features influenced investors' actions even when information about trustees' past behavior was available; however the trustworthiness premium was reduced to 6%.

The stimuli presented in our studies were tightly controlled for all variable facial features. Therefore, and in contrast to most previous studies documenting the economic benefits of a trustworthy appearance, our experiments directly link these benefits to stable facial features. These features are particularly interesting because they are generally impossible to fake and unfakeable cues to trustworthiness are more likely to be reliable than adjustable ones (such as hairstyle, glasses, etc.). We do not claim that unfakeable facial configurations are actually diagnostic of trustworthiness, or even that any diagnostic facial cues to trustworthiness exist. We argue instead that if such cues were to be found, economic theory suggests they should be difficult to simulate. Otherwise, all individuals interested in appearing trustworthy, regardless of their true intentions, could mimic them, thereby limiting the informational value of such cues.

These results do not completely rule out the possibility that the interpretation of stable facial features in our stimuli may partly be related to the reading of subtle emotional expressions. Indeed, when people are asked to place the untrustworthy and trustworthy identities on an angry-to-happy scale, they judge the trustworthy ones to be happier: in a follow-up study, not reported here, we found that the average score for trustworthy faces was 5.9 on a scale from 1 = “angry” to 7 = “happy”, while the average score for untrustworthy faces was 3.6; t(15) = 10.91, p<.001. Note, however, that the artificial nature of this rating task prompts people to think of these faces in terms of “happy” or “angry” labels. Using a different task, which asked participants to categorize faces produced by the trustworthiness computer model according to one of six basic emotions or as being neutral, Todorov and colleagues found that faces falling within 3 standard deviations of the middle point on the trustworthy dimension (like the ones used in the current study) were perceived as emotionally neutral [18]. Thus, while some facial configurations used in our study may resemble emotional expressions, the relation is very subtle and, most importantly, corresponds to the way natural faces are perceived in reality: some of the stable features of natural faces do look, for example, slightly angry or slightly happy, even when these faces are “at rest” (i.e., not expressing any emotions).

This paper focused on unfakeable facial features and their impact on economic behavior, in a controlled environment with different degrees of information. The holistic nature of our stimuli manipulations does not allow us to establish which specific facial features drive the face trustworthiness effect; this is an interesting question for future studies. Future research might also explore the accuracy of face-based trustworthiness impressions. The main challenge for studies claiming the validity of these impressions will be to identify plausible mechanisms that could explain any observed correlations between actual trustworthiness and facial structure (e.g., hormones [22]). For now, reliance on faces to infer trustworthiness seems to favor (perhaps unfairly) those who happen to possess the ‘right’ facial structure.

## Materials and Methods

The current work was approved by the UCL Research Ethics Committee. All participants gave written informed consent before starting the experiment.

### Study 1

#### Participants

Eighteen individuals participated for payment (£4 show-up fee plus variable bonus, see Procedure below). Three were excluded from our analysis: one because of technical problems (malfunctioning camera) and two who conceded to having decided (before starting the experiment) on a strategy to always invest the whole amount available. We thus analyzed data from 15 participants (four female, age range = 18–69 years, Median = 25 years).

#### Face stimuli

**Figure 2:**
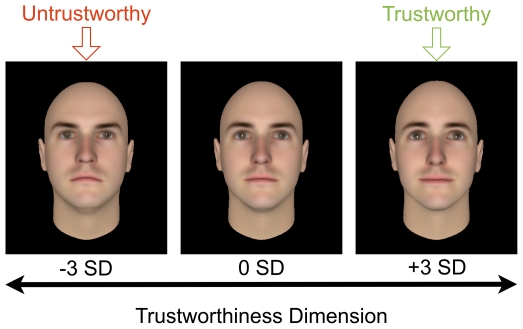
Examples of face stimuli. Face identities of the same computer character varied on the trustworthiness scale. For each character, we selected the faces found at −3 and +3 SD on the trustworthiness scale (indicated here with arrows). In Study 3, we altered some of the selected faces to ensure direct gaze for all stimuli.

We used 40 of the computer-generated faces employed in a previous paper (see Study 5 of [12]), which developed a computer model that manipulates faces to make them less or more trustworthy-looking. Twenty Caucasian faces (‘characters’) with neutral expressions were generated randomly using Facegen software (www.facegen.com). For each character, the model produced two different facial ‘identities’ at opposing ends of the trustworthiness scale (at −3 and +3 SD away from the original face on the model's trustworthiness dimension; see Figure 2). The distance on the trustworthiness scale between the identities was sufficiently large that participants would be unlikely to realize the two identities were derived from the same face, but not so extreme that faces lost their neutral expression [18] or looked unrealistic. Using untrustworthy and trustworthy identities of the same character allowed us to directly measure the effects of stable facial features linked to a trustworthy appearance.

#### Procedure

Participants were ostensibly engaged in a series of online trust games [13] and all were assigned to the role of investor. On each round, they received 100 virtual pounds (VP) and could invest any part of this amount in a trustee whose computerized face appeared on the screen (the face stimuli described above). The amount invested tripled before reaching the trustee. Participants were (falsely) told the trustees were real players from other universities who could decide, without any obligation, to return part of the tripled amount to the investors. Furthermore, participants were (correctly) informed that they would be paid based on their accumulated earnings across 40 rounds of the game (according to an exchange rate of £1 per 1000 VP) so they had an incentive to invest in trustees who would return more than their initial investment. Thus, the amount invested in each partner measured the perceived trustworthiness associated with the corresponding face identity. We stressed the anonymity of the game and that interactions were non-repeating (i.e., only one interaction with each trustee). There was no time limit for decisions, nor feedback provided after each round; the amounts ‘returned’ by trustees were concealed to avoid subsequent decisions being affected by earlier outcomes. The facial stimuli were presented in random order, with the constraint that the two (trustworthy and untrustworthy) face versions of the same character could not be presented directly one after the other.

We took a number of measures to ensure participants believed they were interacting with real trustees. First, we insisted participants arrive on time for the experiment so that they could start at the (allegedly) agreed-upon time with their partners in the game. If they arrived more than five minutes late (or failed to show up), we rescheduled the experiment at a later date. Second, before starting the experiment, participants were photographed wearing a neutral expression and their photo was uploaded into Facegen to create a “computerized” version of their face. These computerized faces were similar to the face stimuli used in our study: they preserved the facial structure of each participant, yet had no hair or specific face identifiers, and had perfect skin texture. After showing participants their own computerized Facegen photo, we pretended to upload it for the trustees to see during the game. Thus, participants had a good reason to believe that the Facegen trustee faces they saw during the experiment were computerized representations of real people's faces whose photos were similarly taken, transformed, and uploaded for the study. Third, between the practice trials and actual games, we intentionally added a delay of several minutes – a fake “waiting time” for other players to (allegedly) join the game – during which the experimenter complained about the difficulties of running such a large scale study. Finally, we added random-length 10–20 second delays between participants' investment decisions and the confirmations they received from trustees (that the latter players' decisions had also been made). This was done to strengthen participants' impressions that they were interacting with real, deliberating human players. Post-experiment interviews confirmed that all participants believed they were interacting in real time with human players and only two participants believed they had seen any character twice (excluding these two participants did not alter the results). This alleviates potential concerns that participants may have been aware of the experimental manipulation (trustworthy vs. untrustworthy faces) and responded accordingly. To avoid contaminating the subject pool, participants were fully debriefed by email only after all testing had been concluded.

### Study 2

#### Participants

Fifty-two participants (30 female, age range: 18–62 years, Median = 23 years) participated for payment (£4 show-up fee plus variable bonus, see Procedure below).

#### Face stimuli

The Study 1 stimuli were used.

#### Behavioral history stimuli

**Figure 3:**
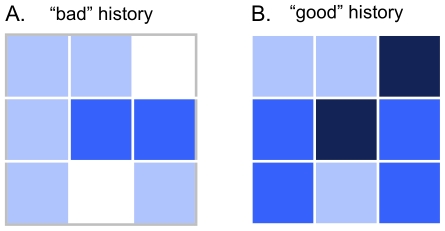
Behavioral history stimuli. Examples of the trustee behavioral histories shown to participants in Study 2. (A) Behavioral history from a “Bad” history trial with two very low (white boxes), five medium-low (light blue), two medium-high (intense blue) and no very high (dark blue) return rates. (B) Behavioral history from a “Good” history trial.

In addition to face identity, this experiment introduced a new variable: each trustee's behavioral history in the trust game. Behavioral histories were presented as 3×3 grids of blue-colored cells varying in shading (Figure 3). Participants were told that these cells represented nine randomly selected return rates in past rounds from the corresponding trustee. Lighter shades of blue corresponded to low return rates and darker shades to high return rates. We used color rather than numbers to avoid explicit arithmetical operations and simple cutoff-rule investment strategies. Our intention was to provide summary representations of partners' behavioral histories. We also aimed to discourage the belief that these histories were perfect predictors of future return rates, hence the random selection and variable nature of trustee past behavior.

The behavioral history variable had two levels: Good and Bad, corresponding to predominantly high and low past return rates, respectively. History stimuli were selected by asking ten volunteers to rate 50 Bad and 50 Good randomly generated behavioral histories (parameters for each condition are available on request) on a scale from 1 (not at all trustworthy) to 7 (very trustworthy). Fifteen histories with average ratings between 2.5 and 3 and the lowest SDs (between 0.47 and 0.95) were picked for the Bad condition; 15 histories with average ratings between 5 and 5.5 and the lowest SDs (between 0.67 and 0.97) were picked for the Good condition. Hence we selected 30 histories that were consistently perceived as either “bad” or “good”, without appearing extreme. These histories were then rotated clockwise and counterclockwise to produce more variations.

#### Experimental design

We had two independent variables of interest: history (Bad vs. Good) and face identity (Trustworthy vs. Untrustworthy). All 40 face identities from Study 1 were used. The total number of trials was 70: 10 trials for each of the 2×2 conditions, plus 30 trials for a control condition where each behavioral history was presented alone (“No-face” condition). The dependent variable was the amount invested by participants.

#### Procedure

The Study 1 procedure was used (including all the steps designed to reduce suspicion concerning the reality of the interactions). In addition, participants were informed that they would not see their partners' faces on some trials.

### Study 3

#### Participants

Twenty participants (13 female, age range: 19–44 years, Median = 21 years) participated for payment. They received a £5 participation fee plus a variable bonus that ranged from £0 to £10, as determined by the outcome of one randomly selected trial (see Procedure below).

#### Face stimuli

The Study 1 stimuli were used. However, in this study, participants saw only one version of each of the 20 Facegen characters, *either* the trustworthy *or* the untrustworthy one, selected at random, with the restriction that each participant be shown 10 trustworthy and 10 untrustworthy faces (for a total of 20 trials). In other words, they saw the trustworthy version of half of the 20 Facegen characters, and the *un*trustworthy version of the other half. Some of the faces were slightly altered to ensure direct gaze.

#### Procedure

Procedure was similar to Study 1 (including all the steps designed to reduce suspicion concerning the reality of the interactions), with a few important changes. Each round, instead of playing with virtual pounds, participants played with 5 real pounds (£), which they could invest (in pence divisions) in the trustees whose faces were displayed on the screen. Participants were told that at the end of the experiment they would be paid a bonus corresponding to the outcome of a single trust game trial that would be randomly selected by the computer. Thus participants had an incentive to approach each round as if it were a one-time trust game with £5 at stake.
